# SIRT6 inhibition delays peripheral nerve recovery by suppressing migration, phagocytosis and M2-polarization of macrophages

**DOI:** 10.1186/s13578-021-00725-y

**Published:** 2021-12-14

**Authors:** Ying Zou, Jiaqi Zhang, Jiawei Xu, Lanya Fu, Yizhou Xu, Xianghai Wang, Zhenlin Li, Lixin Zhu, Hao Sun, Hui Zheng, Jiasong Guo

**Affiliations:** 1 Department of Histology and Embryology, School of Basic Medical Sciences, Southern Medical University, Guangzhou, 510515 China; 2 Guangdong Provincial Key Laboratory of Construction and Detection in Tissue Engineering, Guangzhou, 510515 China; 3 Department of Spine Orthopedics, Zhujiang Hospital, Southern Medical University, Guangzhou, 510280 China; 4 Bioland Laboratory (Guangzhou Regenerative Medicine and Health Guangdong Laboratory), Guangzhou, 510700 China; 5 Key Laboratory of Mental Health of the Ministry of Education, Guangdong Province Key Laboratory of Psychiatric Disorders, Guangdong-Hong Kong-Macao Greater Bay Area Center for Brain Science and Brain-Inspired Intelligence, Guangzhou, 510515 China

**Keywords:** SIRT6, Peripheral nerve injury, Macrophages, Migration, Phagocytosis, Polarization

## Abstract

**Background:**

Silent information regulator 6 (SIRT6) is a mammalian homolog of the nicotinamide adenine dinucleotide (NAD)-dependent deacetylase sirtuin family. Prior evidences suggested that the anti-inflammatory function of SIRT6 after spinal cord and brain injury, and it plays a crucial role in macrophages polarization of adipose tissue and skin. However, the role of SIRT6 in macrophages involved peripheral nerve injury is still unknown. Given the prominent role of macrophages in peripheral nerve recovery, we aim to investigate the role of SIRT6 in the regulation of phenotypes shift and functions in macrophages after peripheral nerve injury.

**Results:**

In the present study, we first identified a significant increase of SIRT6 expression during nerve degeneration and macrophages phagocytosis. Next, we found nerve recovery was delayed after SIRT6 silencing by injected shRNA lentivirus into the crushed sciatic nerve, which exhibited a reduced expression of myelin-related proteins (e.g., MAG and MBP), severer myoatrophy of target muscles, and inferior nerve conduction compared to the shRNA control injected mice. In vitro, we found that SIRT6 inhibition by being treated with a selective inhibitor OSS_128167 or lentivirus transfection impairs migration and phagocytosis capacity of bone marrow-derived macrophages (BMDM). In addition, SIRT6 expression was discovered to be reduced after M1 polarization, but SIRT6 was enhanced after M2 polarization in the monocyte-macrophage cell line RAW264.7 and BMDM. Moreover, SIRT6 inhibition increased M1 macrophage polarization with a concomitant decrease in M2 polarization both in RAW264.7 and BMDM via activating NF-κB and TNF-α expression, and SIRT6 activation by UBCS039 treatment could shift the macrophages from M1 to M2 phenotype.

**Conclusion:**

Our findings indicate that SIRT6 inhibition impairs peripheral nerve repair through suppressing the migration, phagocytosis, and M2 polarization of macrophages. Therefore, SIRT6 may become a favorable therapeutic target for peripheral nerve injury.

## Introduction

In the injured peripheral nerve, the axons and myelin will undergo fragmentation and degeneration and the debris have to be cleared by phagocytosis of macrophages and Schwann cells [1, 2]. Prior evidences suggested that suppressed recruitment of macrophages into the injured site or depletion of macrophages impairs nerve regeneration and results in a worse outcome [3, 4]. Macrophages can be polarized into two subpopulations: classically activated M1 macrophages and alternatively activated M2 macrophages [5], and the phenotypes will shift in the various stages of nerve repair [6, 7]. Traditionally, M1 macrophages are associated with pro-inflammatory function and neurodegenerative outcome, whereas M2 macrophages are broadly known as anti-inflammatory cells for promoting nerve repair [7]. In short, the migration, phagocytosis and polarization of macrophages play important roles in nerve regeneration.

Silent information regulator 6 (SIRT6) is a mammalian homolog of the nicotinamide adenine dinucleotide (NAD)-dependent deacetylase sirtuin family [8]. SIRT6 has been reported involving in transcriptional regulation, genome stability and longevity in many kinds of cells [9, 10]. Recently, reports mentioned the roles of SIRT6 in macrophages, such as adipocyte-specific SIRT6 deficiency increases macrophages infiltration and IL-4 production in white adipose tissue, which in turn affects M2 macrophage polarization [11], and myeloid cell-specific SIRT6 deficiency delays skin wound healing in mice by modulating inflammation and decreases M2 phenotypic switching [12]. However, the outcome of SIRT6 inhibition specifically in macrophages as well as its roles in peripheral nerve injury are still unclear.

Herein, we aim to reveal some questions: (1) does SIRT6 inhibition affects nerve regeneration and functional recovery? (2) the potential role of SIRT6 in migration, phagocytosis and polarization of macrophages in the peripheral nerve? Overall findings of the present study suggested that SIRT6 regulates migration, phagocytosis and M1/M2 polarization, thus affects functional recovery and nerve regeneration after peripheral nerve injury.

## Results

### SIRT6 expression is up-regulated and accompanied with macrophages infiltration in the transected sciatic nerve

**Fig. 1:**
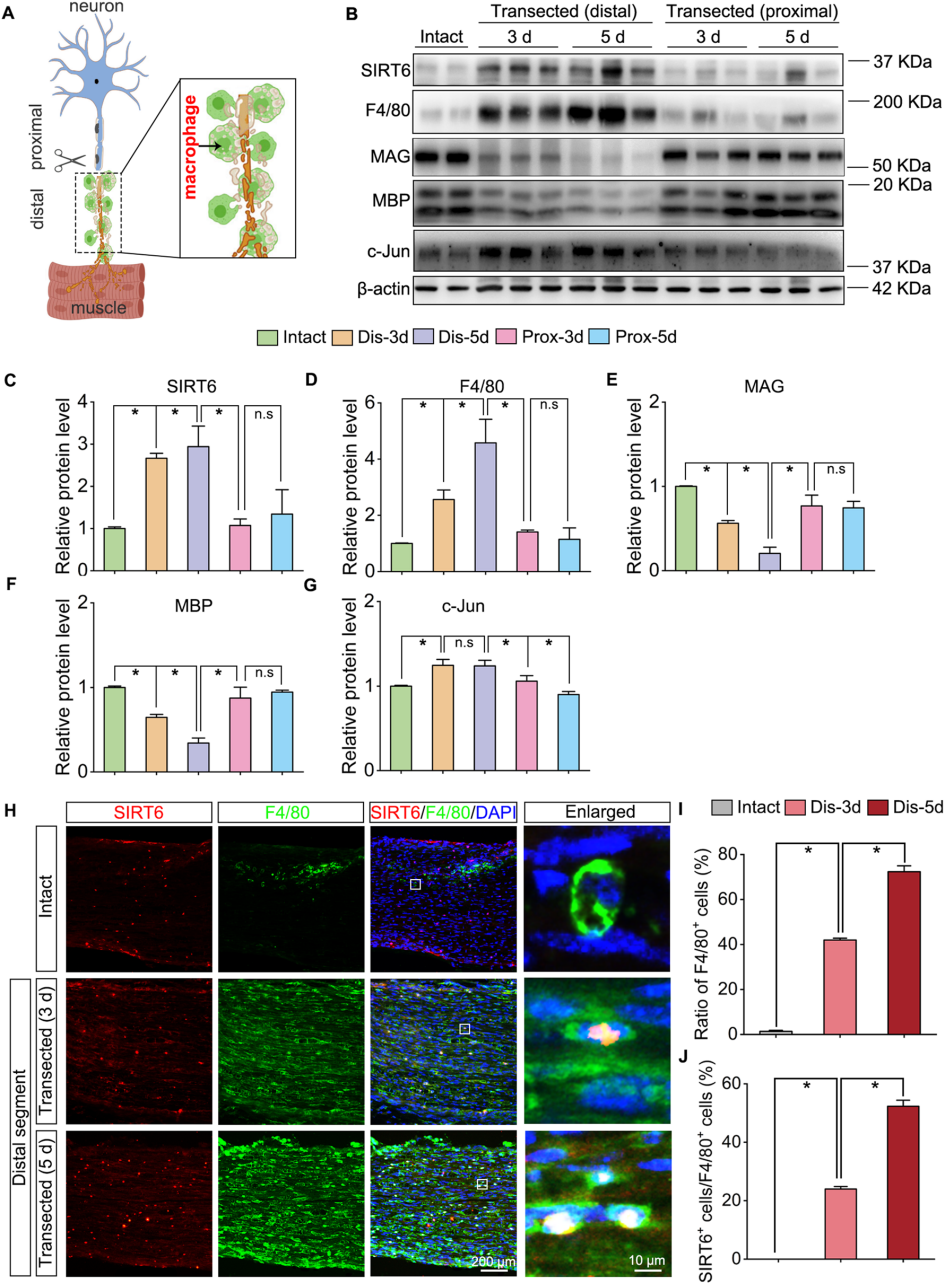
Increased expression of SIRT6 and accompanied with macrophages infiltration in the transected sciatic nerve. **A** Schematic diagram of macrophages infiltration in the distal of injured nerve. The black arrow points to the macrophages. **B** Western blots of SIRT6, F4/80, MAG, MBP, and c-Jun expression in the distal and proximal stump of the transected sciatic nerve, the intact sciatic nerve was used as the control. **C**–**G** Quantification of SIRT6, F4/80, MAG, MBP, and c-Jun protein levels in the blots. The protein levels were normalized with β-actin. n.s: non-significance; *P  < 0.05. **H** Immunofluorescence images showing the F4/80 positive macrophages (green) infiltrates the distal stump of the injured sciatic nerves and SIRT6 (red) expression is up-regulated in the macrophages in a time-dependent manner after transection. Scale bar:200 µm, 10 µm in the enlarged parts. **I** Quantification of F4/80 positive cells ratio (F4/80^+^/DAPI^+^). **J** Quantification of SIRT6^+^/F4/80^+^ cells ratio. *P  < 0.05. *Dis* distal; *Prox* proximal

As well known, when axons are injured by nerve transection or nerve crush, the distal nerve segment fragments and degenerates, eventually myelin and axonal debris are predominantly cleared by macrophages (Fig. 1A) [1]. To determine whether SIRT6 is related to macrophages infiltration, we examined the SIRT6 and F4/80 expression as well as expression of myelin-related proteins (e.g., MAG, MBP, and c-Jun) by Western blotting. A significantly increased of SIRT6 expression was found in the distal stump of transected sciatic nerve instead of in the proximal stump, when compared with the intact nerve (Fig. 1B, C). And interestingly, SIRT6 expression was accompanied with F4/80 expression which indicates macrophages infiltration (Fig. 1B–D). Furthermore, the expression of MAG and MBP was down-regulated in the distal segments of nerves, whereas c-Jun (known to be a key transcription factor of nerve injury response [13]) was significantly increased after transection (Fig. 1B, E–G). Furthermore, immunofluorescence analysis showed that few numbers of macrophages (identified as F4/80-positive) were present and SIRT6 expression was very weak in the intact sciatic nerve (Fig. 1H). Differently, the increased SIRT6 staining was found and accompanied with F4/80 staining within the transected sciatic nerves at 3-days and 5-days post injury in a time-dependent manner (Fig. 1H–J).

### SIRT6 silencing by shRNA lentivirus transfection impairs peripheral nerve recovery

**Fig. 2:**
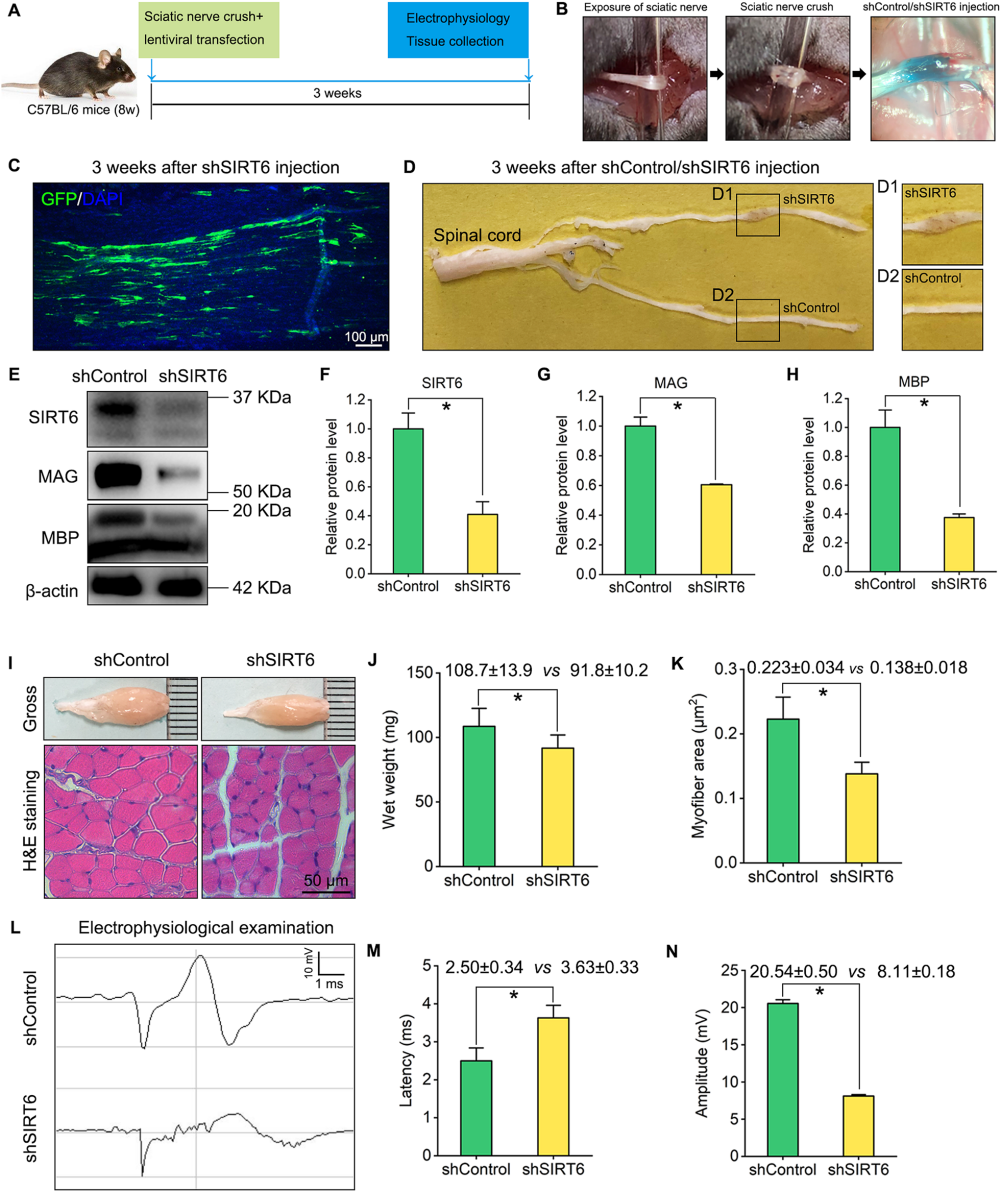
SIRT6 silencing in crushed sciatic nerve delays nerve and functional recovery. **A** Flow path of shControl or shSIRT6 lentivirus microinjection in crushed sciatic nerve of mice, the mice were received electrophysiology examination and tissue collection for Western blotting and histochemistry analysis after 3 weeks. **B** Gross image of shControl/shSIRT6 microinjection after nerve crush immediately. The aquamarine blue indicates fast green. **C** An intense GFP (green) labeling in the lentivirus injected sciatic nerve. Scale bar: 100 µm. **D** Compared with shControl-injected nerve, shSIRT6-injected nerve reveals more redness and swelling, indicates a severer inflammation response. **E** Western blotting of SIRT6, MAG and MBP in the shControl/shSIRT6 injected sciatic nerves at 3 weeks post-surgery. **F**–**H** Quantification of SIRT6, MAG and MBP levels in the blots. The protein levels were normalized with β-actin. *P  < 0.05. **I** Gross image and H&E staining of gastrocnemius muscles. Scale bar: 50 µm. **J**, **K** Quantification of wet weight and myofiber area of gastrocnemius muscles. *P  < 0.05. **L** Electrophysiological examination of the compound action potential in the shControl/shSIRT6-injected sciatic nerves. **M**, **N** Quantification of latency (ms) and potential amplitude (mV). *P  < 0.05

To investigate the role of SIRT6 in peripheral nerve recovery, lentiviral vector encoding SIRT6 shRNA (shSIRT6) that efficiently down-regulating SIRT6 expression was micro-injected into crushed sciatic nerve (Fig. 2A, B). Three weeks later, lentivirus injected nerve showed an intense GFP (green) labelling (Fig. 2C). From the gross morphology, the shSIRT6 lentivirus injected nerve had more redness and swelling than the contralateral nerve which was injected with shControl lentivirus (Fig. 2D), which hint that shSIRT6-injected sciatic nerve is undergoing a more serious inflammation response. By Western blotting, the results showed SIRT6 expression was effectively decreased in the shSIRT6 injected nerve and the MAG/MBP expression levels in shSIRT6 injected nerve were weaker than those in shControl injected nerve (Fig. 2E–H). Next, we assessed the wet weight and the average myofiber area of gastrocnemius, a key target muscle of the sciatic nerve, as these measures are widely used to assess nerve regeneration [14, 15]. Gross observation of the gastrocnemius and H&E staining in the cross sections showed that myoatrophy was severer in the shSIRT6 injected group compared with the shControl group (Fig. 2I–K). Then, electrophysiological examination was evaluated to assess nerve conduction (Fig. 2L). Statistical analysis showed that, compared with the shControl group, shSIRT6 injection results in a longer latency (2.50 ± 0.34 ms vs. 3.63 ± 0.33 ms, which corresponds to slower nerve conduction[16]) as well as an inferior amplitude (20.54 ± 0.50 mV vs. 8.11 ± 0.18 mV, which indicates that less axons and myelin regenerated [16]) (Fig. 2M, N).

### SIRT6 inhibition suppresses migration of macrophages

**Fig. 3:**
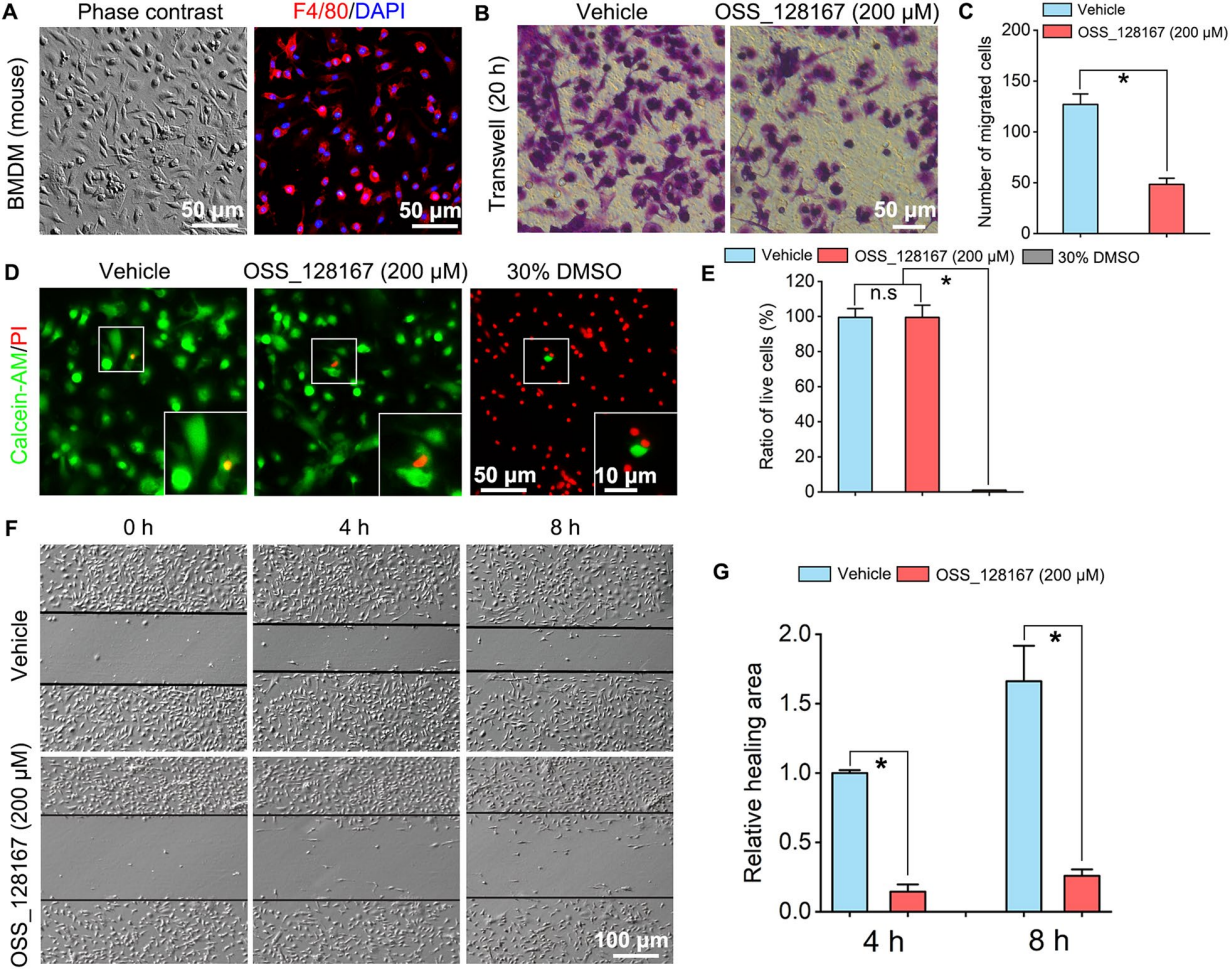
SIRT6 inhibition suppresses macrophages migration*.* **A** Culture and identification of bone marrow-derived macrophages (BMDM). Left: phase contrast microscopy of BMDM; Right: immunofluorescence of macrophages maker F4/80 staining. Scale bar: 50 µm. **B** Transwell assay: the migrated macrophages in the Vehicle group and OSS_128167 treated group. Scale bar: 50 µm. **C** Quantification of migrated macrophages number, the number of migrated macrophages in OSS_128167 treated group is significantly decreased compared with that in the Vehicle group. *P  < 0.05. **D** Live/dead staining of BMDM treated with OSS_128167 (200 μM) after 24 h. The live cells were stained by Calcein-AM with green and the dead cells were stained by PI with red. Scale bar: 50 µm, 10 µm in the enlarged parts. **E** Quantification of live cells ratio, the ratio of live cells in the OSS_128167 group were similar to the Vehicle group. n.s: no significance; *P  < 0.05. **F** Scratch assay: after 4 h and 8 h, more slower gap closure (or healing area) was seen with BMDM of treated with OSS_128167. **G** Quantification of the relative healing area of scratch. *P  < 0.05. OSS_128167, SIRT6 inhibitor

Considering SIRT6 expression is up-regulated and accompanied with macrophages infiltration in the injured nerve, and macrophages play a vital role in nerve regeneration, further investigations were designed to reveal the effects of SIRT6 inhibition on the main biofunctions of macrophages including migration, phagocytosis and polarization to understand the potential mechanisms of macrophages in the SIRT6 inhibition delaying nerve repair. Macrophages were isolated from the mice bone marrow and identified by F4/80 staining (Fig. 3A). Transwell assay was performed to investigate the effect of SIRT6 inhibition with OSS_128167 treatment on macrophages migration, which illustrated that much less migrated macrophages were detected in the OSS_128167 group when compared with Vehicle group (Fig. 3B, C). To exclude the bioeffects of potential cytotoxicity of OSS_128167 for the migration assay, the live/dead staining was performed and the result illustrated that overwhelming majority of cells were survived (positive for green Calcein-AM) after cultured with OSS_128167, while the cells treated with 30% DMSO for the positive control illustrated overwhelming majority of cells were died (positive for red PI) (Fig. 3D, E). Furthermore, scratch assay was also performed on the cultured BMDM and the result showed, slower gap closure (or healing area) was seen in the OSS_128167 group (Fig. 3F, G). These results demonstrate that SIRT6 inhibition suppresses macrophages migration.

### SIRT6 expression is increased during macrophages phagocytosis and SIRT6 inhibition suppresses phagocytosis of macrophages

**Fig. 4:**
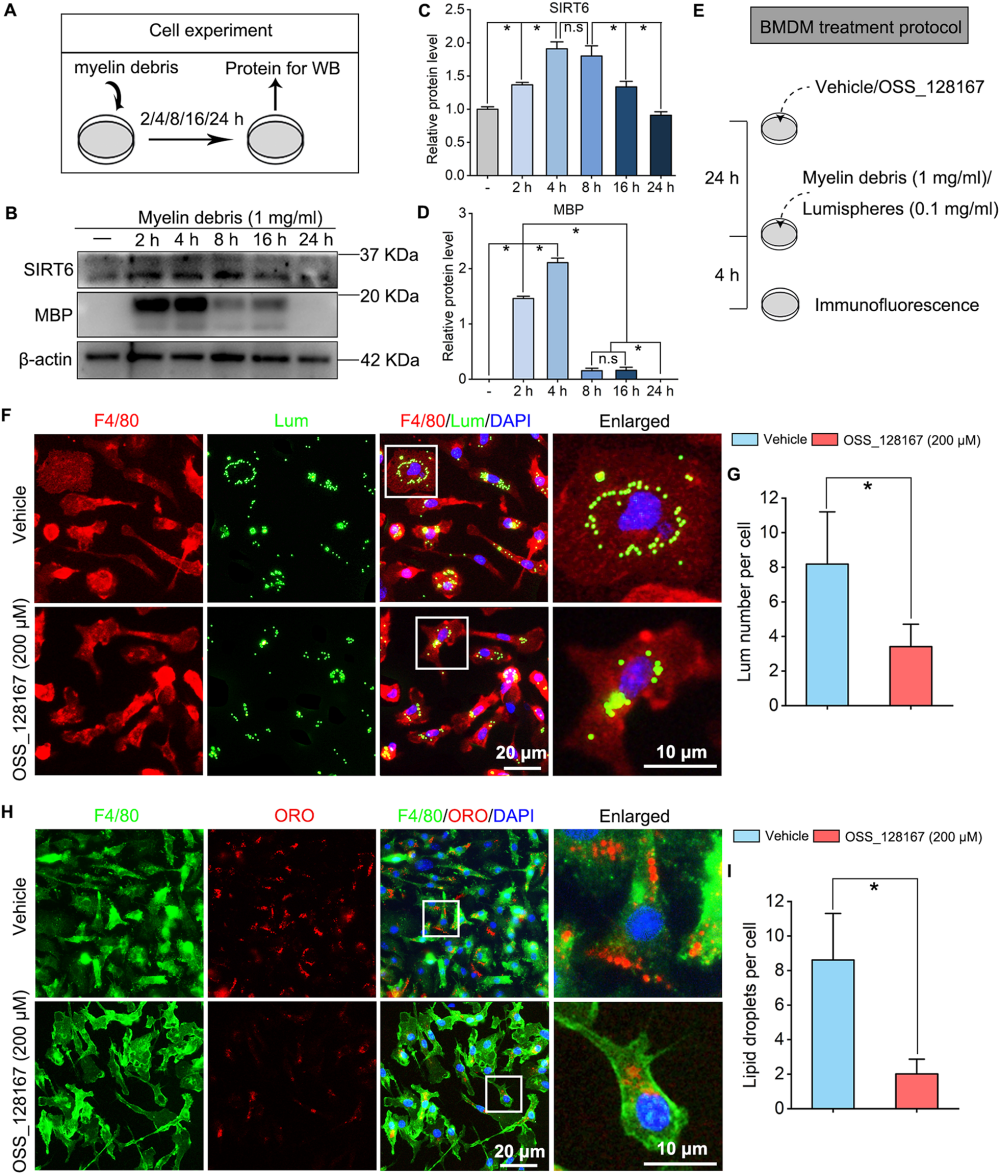
SIRT6 expression is increased during macrophages phagocytosis and SIRT6 inhibition suppresses phagocytosis of macrophages. **A** Cell experiment protocol of macrophages phagocytosis. **B** Western blots of SIRT6, and MBP. **C**, **D** Quantification of SIRT6, and MBP protein levels in the blots. n.s: no significance; *P  < 0.05. **E** Protocol of BMDM treated with Vehicle or OSS_128167 (200 μM) for 24 h, and then 1 mg/ml myelin debris or 0.1 mg/ml fluorescence lumispheres (green) was added into the cultures for 4 h. **F** Double immunofluorescence staining for F4/80 stained macrophages and fluorescence lumispheres. Scale bar: 20 µm, 10 µm in the enlarged parts. **G** Quantification of the lumispheres number per macrophage. *P  < 0.05. **H** Double of immunofluorescence staining for phagocytosis of F4/80 stained macrophages and myelin debris (stained by ORO). Scale bar: 20 µm, 10 µm in the enlarged parts. **I** Quantification of lipid droplets per macrophage. *P  < 0.05. OSS_128167, SIRT6 inhibitor

Next, we assessed whether SIRT6 inhibition alters phagocytosis of macrophages. As shown in the experiment protocol, 1 mg/ml myelin debris was added in the cell medium and the BMDM were collected for protein extraction after 2, 4, 8, 16 and 24 h, respectively (Fig. 4A). We identified a significant increase of SIRT6 expression in macrophages during myelin debris incubation, suggest that SIRT6 might take part in the process of phagocytosis. Besides, MBP expression is represented for the content of myelin debris in macrophages, which reaches the peak at 4 h (Fig. 4B–D). Therefore, the myelin debris was incubated for 4 h in the following experiments. To examine the effect of SIRT6 inhibition on phagocytosis of macrophages, 1 mg/ml myelin debris or 0.1 mg/ml fluorescence lumispheres (green) was added into the OSS_128167 or vehicle treated BMDM for 4 h (Fig. 4E). Double immunofluorescence illustrated that the number of phagocytosed lumispheres per cell in the OSS_128167 group was less than those in the Vehicle group (Fig. 4F, G). Meanwhile, Oil red O (ORO) staining showed a significant reduction of myelin debris in the OSS_128167 treated cells (Fig. 4H, I).

### Lentivirus-mediated SIRT6 silencing inhibits phagocytosis of macrophages

**Fig. 5:**
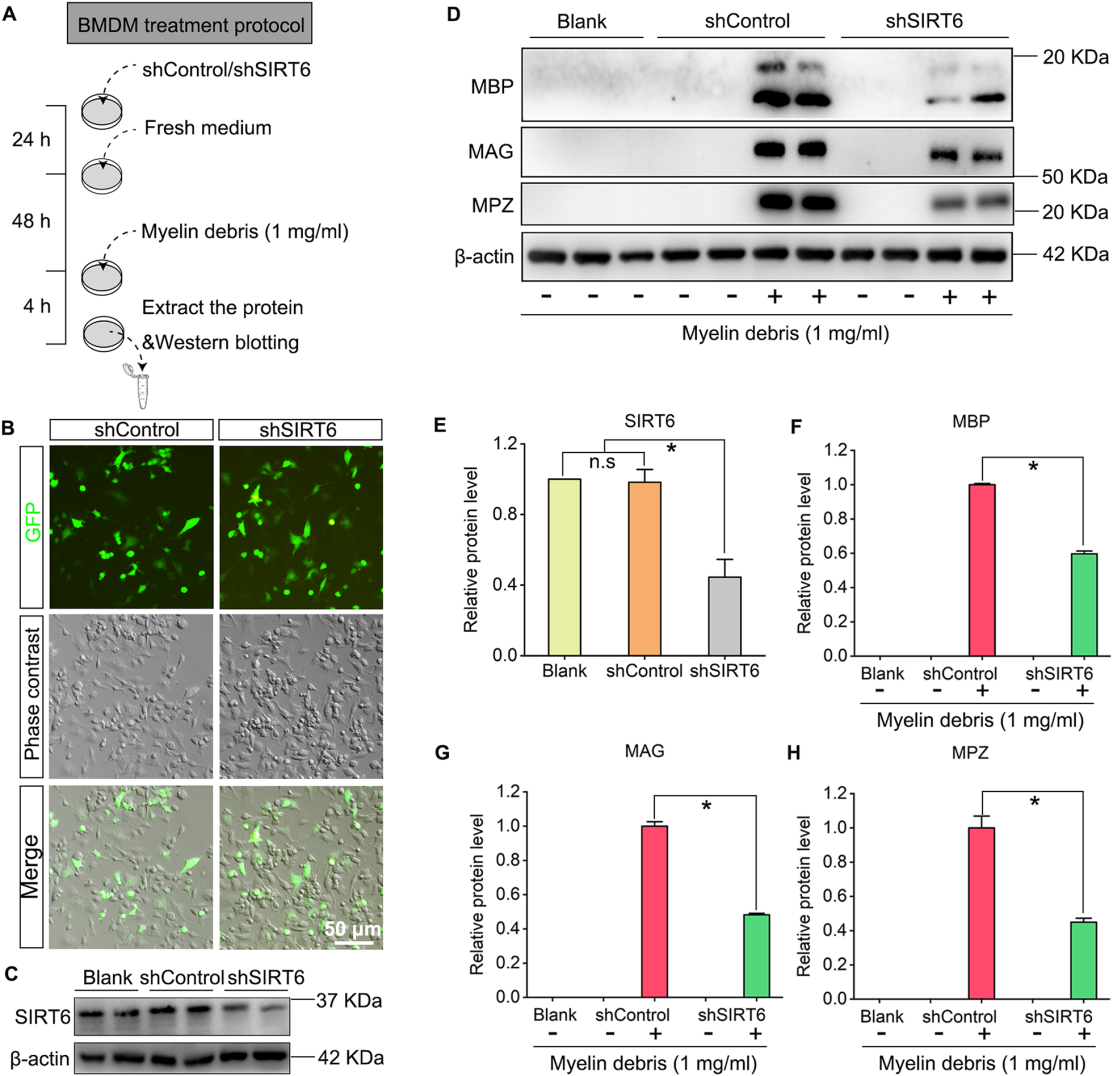
shSIRT6 transfection silences SIRT6 expression and inhibits phagocytosis of macrophages. **A** BMDM was transfected with shControl or shSIRT6 lentivirus for 24 h and cultured for further 48 h, then 1 mg/ml myelin debris was added into the cultures and the cells were collected for Western blotting after 4 h. **B** GFP transfection efficiency was quantitatively evaluated by fluorescence microscopy. Scale bar: 50 µm. **C** Western blot of SIRT6 in the blank BMDM, and shControl or shSIRT6 transfected BMDM. **D** Western blots of MBP, MAG and MPZ in the shControl or shSIRT6 transfected BMDM and incubated with myelin debris. **E**–**H** Quantification of SIRT6, MBP, MAG and MPZ protein levels in the blots. The protein levels were normalized with β-actin. n.s: no significance; *P  < 0.05. “−” indicates without myelin debris; “+” indicates myelin debris addition

To further confirm the role of SIRT6 in macrophages phagocytosis, the shRNA lentivirus transfection was performed to knock down SIRT6 expression in macrophages (Fig. 5A). Report gene GFP expression indicates high transfection efficiency (Fig. 5B). Western blotting analysis showed shSIRT6 lentivirus effectively decrease the SIRT6 expression in macrophages (Fig. 5C, E). And the myelin debris co-culture assessment indicated shSIRT6-transfection also alleviates the phagocytosis function of BMDM, characterized by a significantly decreased expression of MBP, MAG and MPZ (Fig. 5D, F–H).

### SIRT6 expression is lower in M1 macrophages but higher in M2 macrophages

**Fig. 6:**
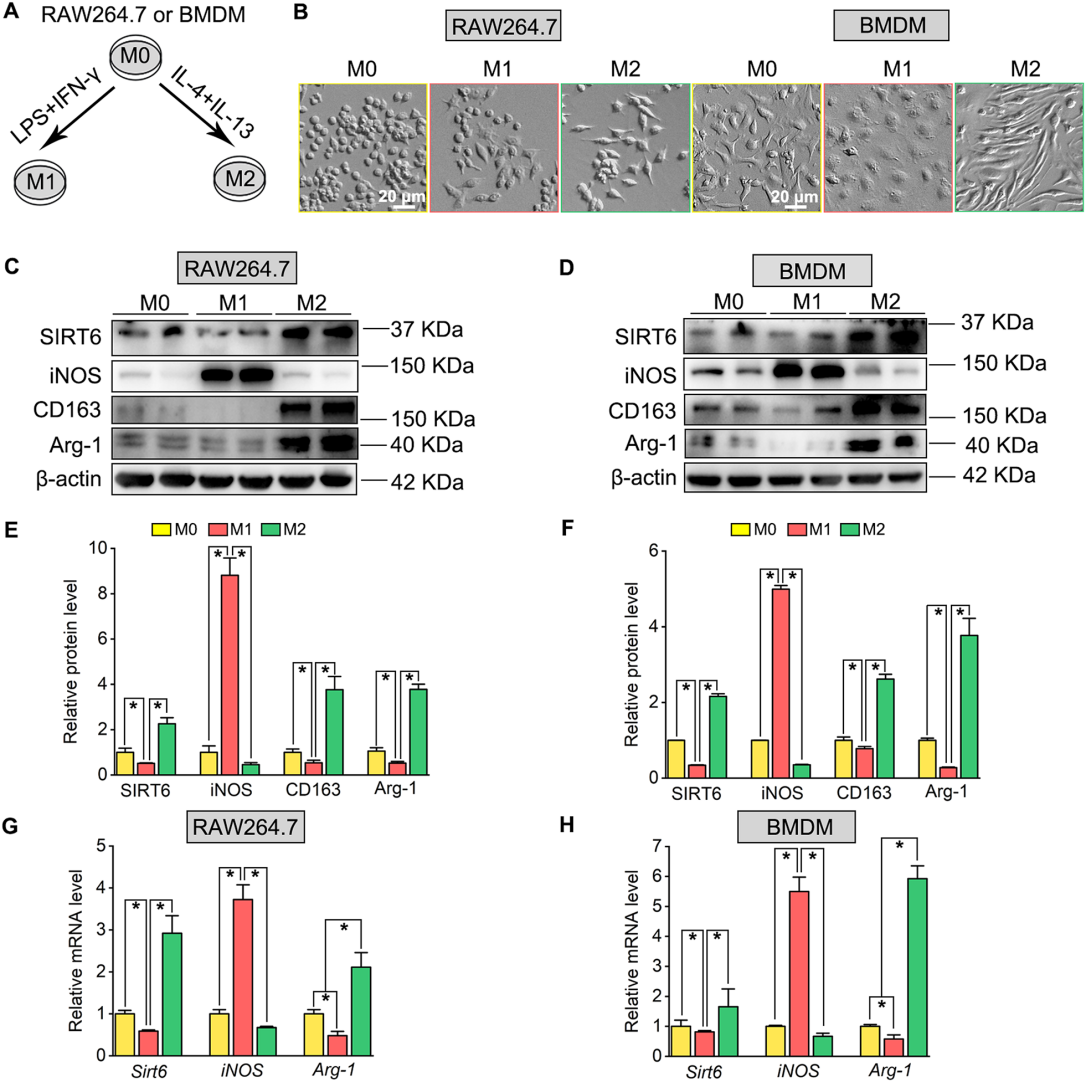
SIRT6 expression is lower in M1 macrophages but higher in M2 macrophages. **A** Protocol of M1 and M2 polarization of macrophages. RAW264.7 and BMDM were induced to M1 type after stimulated with lipopolysaccharide (LPS) and IFN-γ, and induced to M2 type after stimulated with IL-4 and IL-13. **B** Phase contrast microscopy of RAW264.7 (left) BMDM (right) after polarization. M0 represents non-induced RAW264.7 or BMDM; M1 represents LPS and IFN-γ treated cells; M2 represents IL-4 and IL-13 treated cells. Scale bar: 20 µm. **C**, **D** Western blots of SIRT6, iNOS, CD163, and Arg-1 expression in the RAW264.7 and BMDM after polarization. **E**, **F** Quantification of SIRT6, iNOS, CD163, and Arg-1 expression in the RAW264.7 and BMDM after polarization. The protein levels were normalized with β-actin. *P  < 0.05. **G**, **H** qPCR analysis of *Sirt6*, *iNOS*, and *Arg-1* mRNA levels in the RAW264.7 and BMDM after polarization. The mRNA level was normalized with GAPDH. *P  < 0.05

To investigate the role of SIRT6 in macrophages polarization, RAW264.7 and BMDM were induced to M1 type after stimulated with lipopolysaccharide (LPS) and IFN-γ, and induced to M2 type after stimulated with IL-4 and IL-13 (Fig. 6A). In terms of cell morphology, M1-polarized macrophages were flattened with irregular shapes, while M2-polarized macrophages were elongated into spindle-shaped, compared to the rounded cell shape in non-induced RAW264.7 or non-induced BMDM (M0) with irregular or spindle shapes (Fig. 6B). Western blotting analysis showed that SIRT6 expression was down-regulated in M1-polarized macrophages manifested as decreased expression of M1 marker iNOS compared with M0 macrophages (Fig. 6C, E), whereas SIRT6 expression was markedly increased in M2-polarized macrophages, characteristics of increased M2 markers including CD163 and Arg-1 expression compared with M0 macrophages (Fig. 6D, F). The SIRT6 mRNA level after polarization was tested by qPCR with the change pattern consists with the results of Western blotting, the SIRT6 mRNA level was markedly decreased in M1-polarized macrophages as well as the increased mRNA level of M1 marker iNOS. Accordingly, the mRNA level of SIRT6 was markedly increased in M2-polarized macrophages, accompanied by the increased mRNA expression of M2 marker Arg-1 (Fig. 6G, H).

### SIRT6 inhibition promotes M1 polarization but suppresses M2 polarization in macrophages via upregulating NF-κB and TNF-α expression

**Fig. 7:**
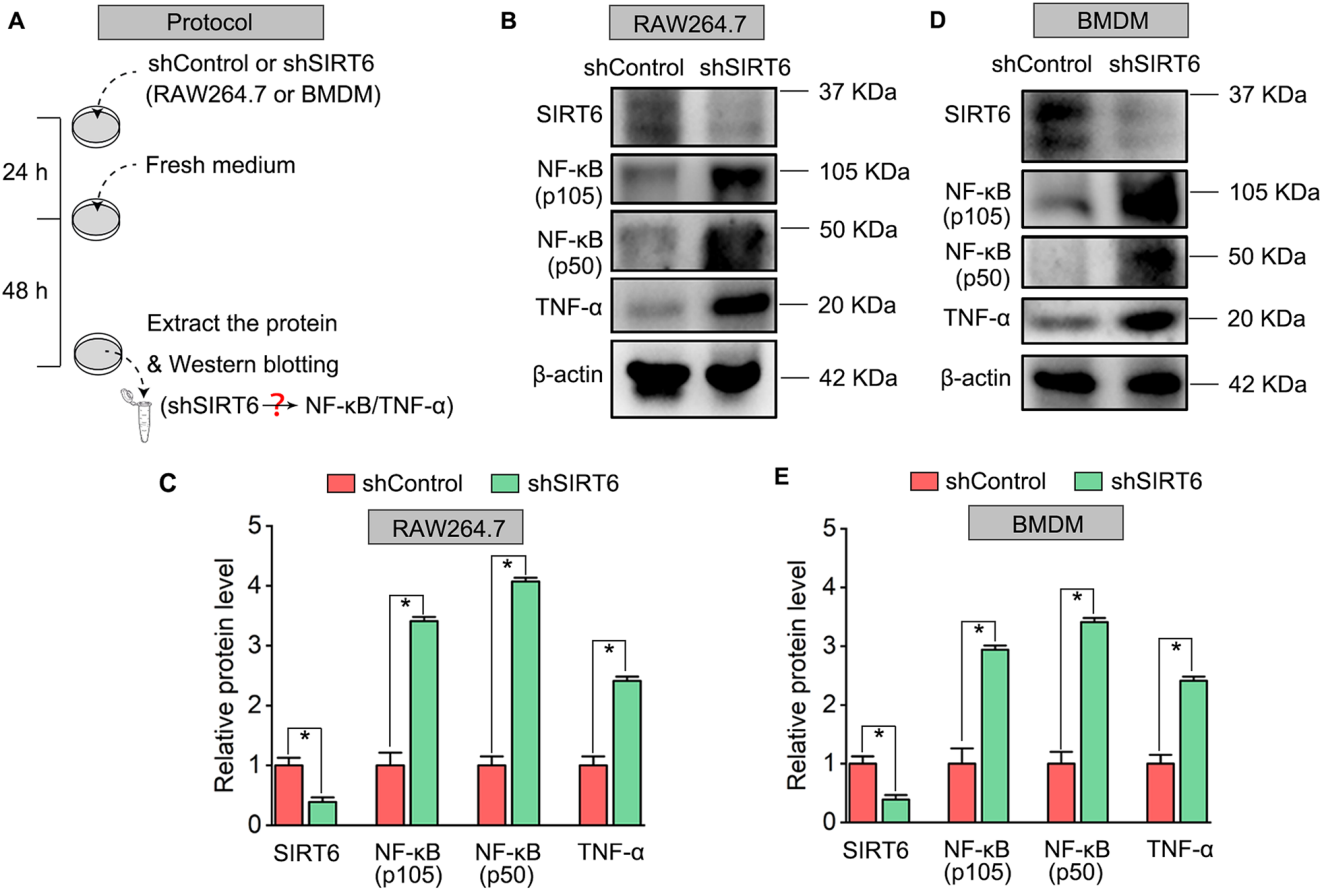
SIRT6 silencing upregulates NF-κB and TNF-α expression in macrophage. **A** shControl or shSIRT6 lentivirus was transfected in RAW264.7 or BMDM for 24 h, the cells were collected and extracted for Western blotting analysis after 48 h. **B**, **D** Western blots of SIRT6, NF-κB, and TNF-α expression in shRNA transfected RAW264.7 or BMDM. **C**, **E** Quantification of SIRT6, NF-κB, and TNF-α protein levels in RAW264.7 and BMDM. The protein levels were normalized with β-actin. *P  < 0.05

**Fig. 8:**
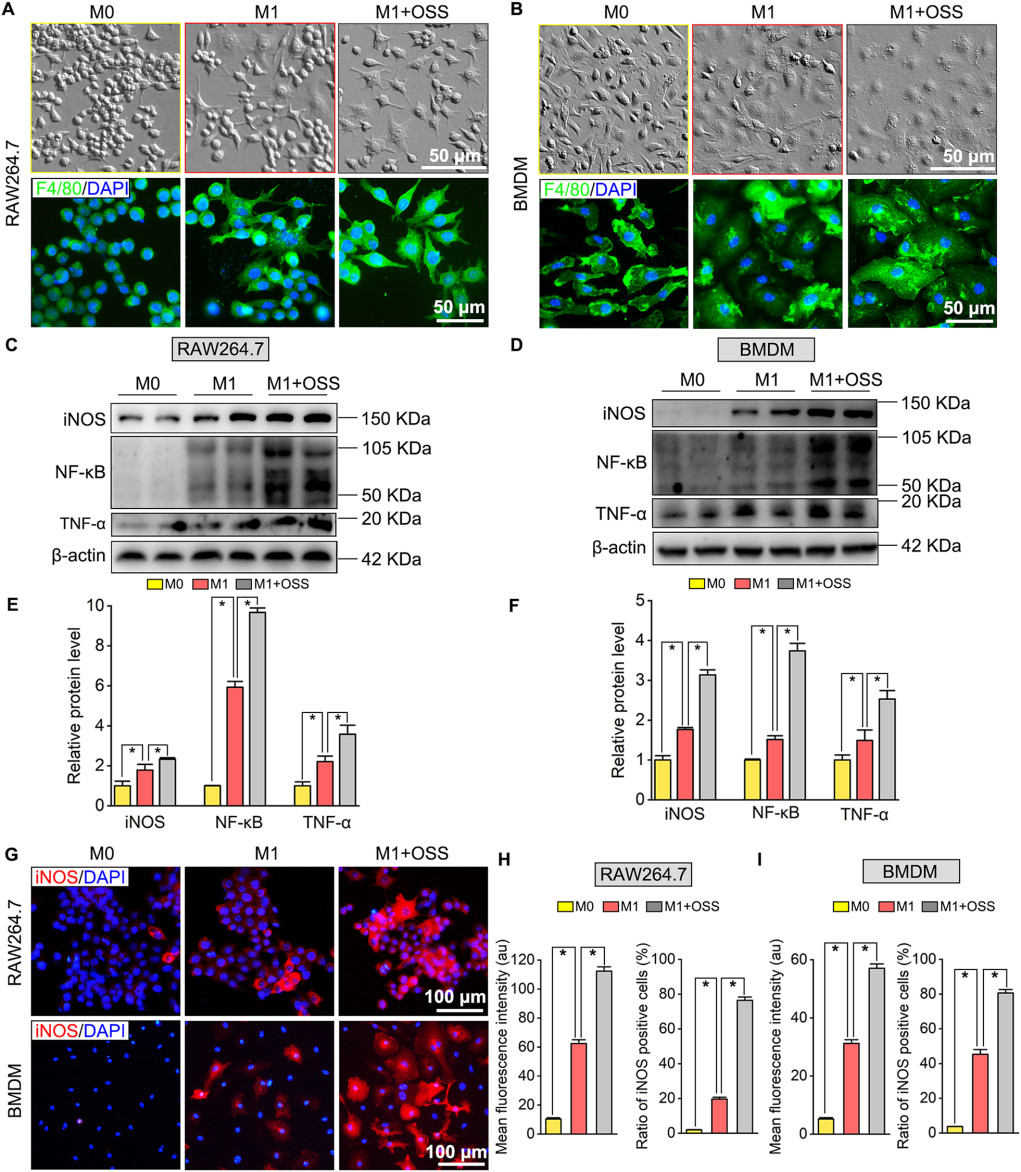
SIRT6 inhibition promotes macrophages polarization toward M1 type. **A**, **B** Cell morphology of RAW264.7 and BMDM after M1 polarization and OSS_128167 treatment. The outlines of RAW264.7 and BMDM were stained by macrophages marker F4/80. Scale bar: 50 µm. **C**, **D** Western blots of iNOS, NF-κB, and TNF-α expression after M1 polarization and OSS_128167 treatment in RAW264.7 and BMDM. **E**, **F** Quantification of iNOS, NF-κB, and TNF-α protein levels in RAW264.7 and BMDM. The protein levels were normalized with β-actin. *P  < 0.05. **G** Immunofluorescence staining for iNOS in RAW264.7 and BMDM. Scale bar: 100 µm. **H**, **I** Quantifications of mean fluorescence intensity (au) of iNOS and ratio of iNOS positive cells (%) in RAW264.7 and BMDM. *P  < 0.05. OSS: OSS_128167, SIRT6 inhibitor

**Fig. 9:**
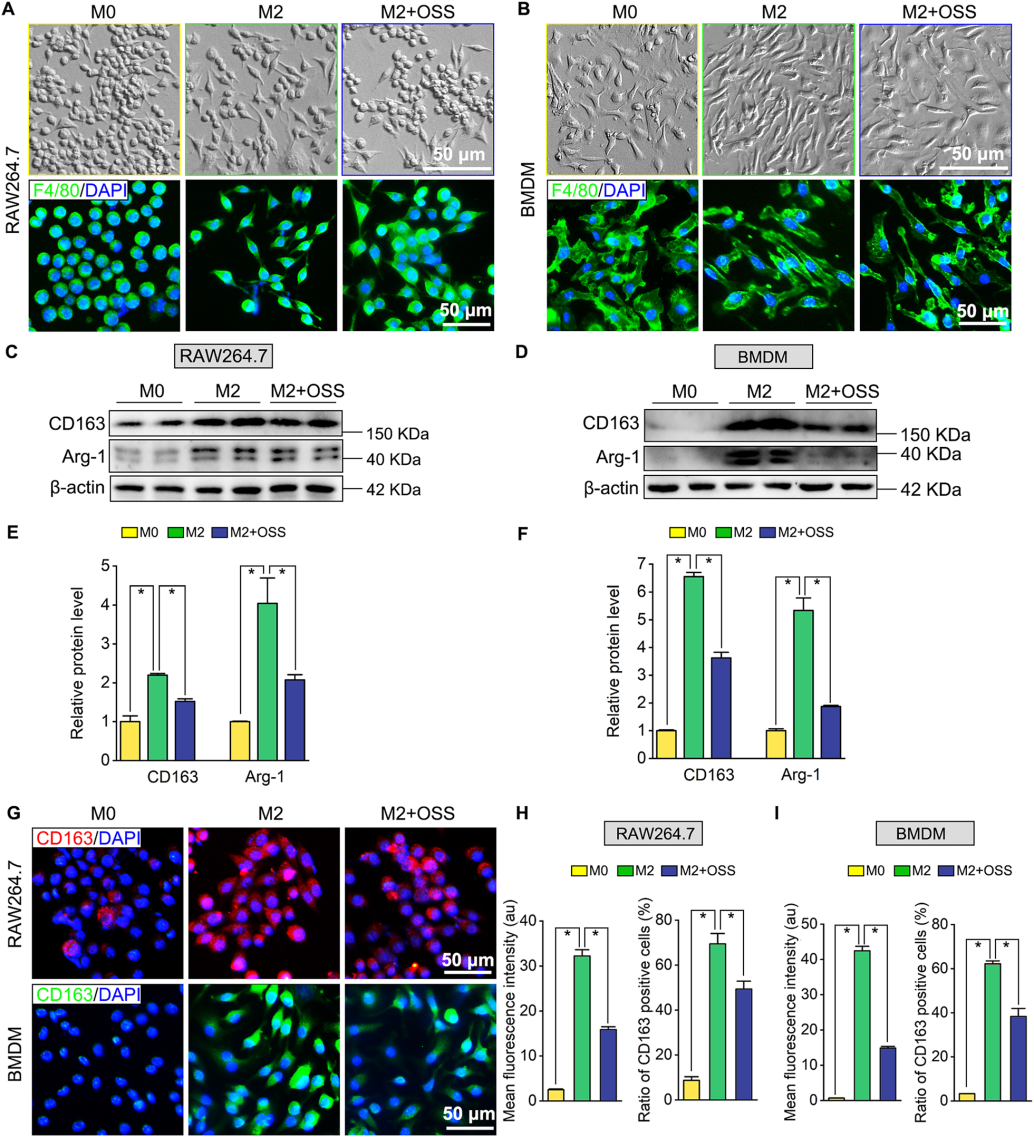
SIRT6 inhibition suppresses macrophages polarization toward M2 type. **A**, **B** Cell morphology of RAW264.7 and BMDM after M2 polarization with OSS_128167 treatment. The outlines of RAW264.7 and BMDM were stained by macrophages marker F4/80. Scale bar: 50 µm. **C**, **D** Western blots of CD163, Arg-1 expression after M2 polarization and OSS_128167 treatment in RAW264.7 and BMDM. **E**, **F** Quantification of CD163 and Arg-1 protein levels in RAW264.7 and BMDM. The protein levels were normalized with β-actin. *P  < 0.05. **G** Immunofluorescence staining for M2 macrophages marker CD163 in RAW264.7 and BMDM. Scale bar: 50 µm. **H**, **I** Quantifications of mean fluorescence intensity (au) of CD163 and ratio of CD163 positive cells (%) in RAW264.7 and BMDM. *P  < 0.05. OSS: OSS_128167, SIRT6 inhibitor

As previously reported, SIRT6 showed a suppression effect on transcription factor NF-κB and pro-inflammatory cytokines TNF-α production and afterwards play vital roles in anti-inflammation [17, 18]. And M1 macrophages are characterized by the secretion of proinflammatory cytokine TNF-α and the activation of NF-κB [19, 20]. Therefore, we examined whether SIRT6 silencing by shRNA lentivirus transfection affects the expression of NF-κB and TNF-α in macrophages (Fig. 7A). Western blotting showed that the marked activation of NF-κB and TNF-α expression after SIRT6 knockdown was found both in RAW264.7 (Fig. 7B, C) and BMDM (Fig. 7D, E). Then, SIRT6 inhibitor OSS_128167 was added into the M1 or M2 polarization medium. In the term of cell morphology, OSS_128167 significantly enhances M1 macrophages features, such as more flattened shape and increased cell number of typical M1 macrophages under phase contrast observation and F4/80 staining, both in RAW264.7 and BMDM (Fig. 8A, B). Next, the expression levels of iNOS, NF-κB and TNF-α were identified by Western blotting, M1-polarized macrophages with OSS_128167 treatment showed an elevated expression of iNOS, NF-κB and TNF-α, compared with those M0/M1 RAW264.7 (Fig. 8C, E) and those M0/M1 BMDM (Fig. 8D, F), respectively. Finally, M1 polarization of macrophages was assessed by immunofluorescence with M1 specific marker iNOS (Fig. 8G). Quantitative results illustrated that the mean fluorescence intensity of iNOS and the ratio of iNOS-positive macrophages were increased dramatically after supplementing with OSS_128167 (Fig. 8H, I), which was consistent with the result of Western blotting. Next, we investigated the effect of SIRT6 inhibition on M2 polarization of macrophages. OSS_128167 resulted in fewer number of M2 typical spindle-shaped cells both in RAW264.7 and BMDM (Fig. 9A, B). Furthermore, Western blotting showed that OSS_128167 decrease the expression of M2 markers (e.g., CD163 and Arg-1) in RAW264.7 (Fig. 9C, E) and BMDM (Fig. 9D, F). Finally, M2 macrophages polarization after OSS_128167 treatment was assessed by immunofluorescence with M2 specific marker CD163. Notably, a decreased expression of CD163 was observed in the macrophages (Fig. 9G). Quantitative results illustrated that the mean fluorescence intensity of CD163 and the ratio of CD163-positive M2 macrophages were decreased significantly after being treated with OSS_128167 both in RAW264.7 and BMDM (Fig. 9H, I).

### SIRT6 activation shifts M1 to M2 phenotype in macrophages

**Fig. 10:**
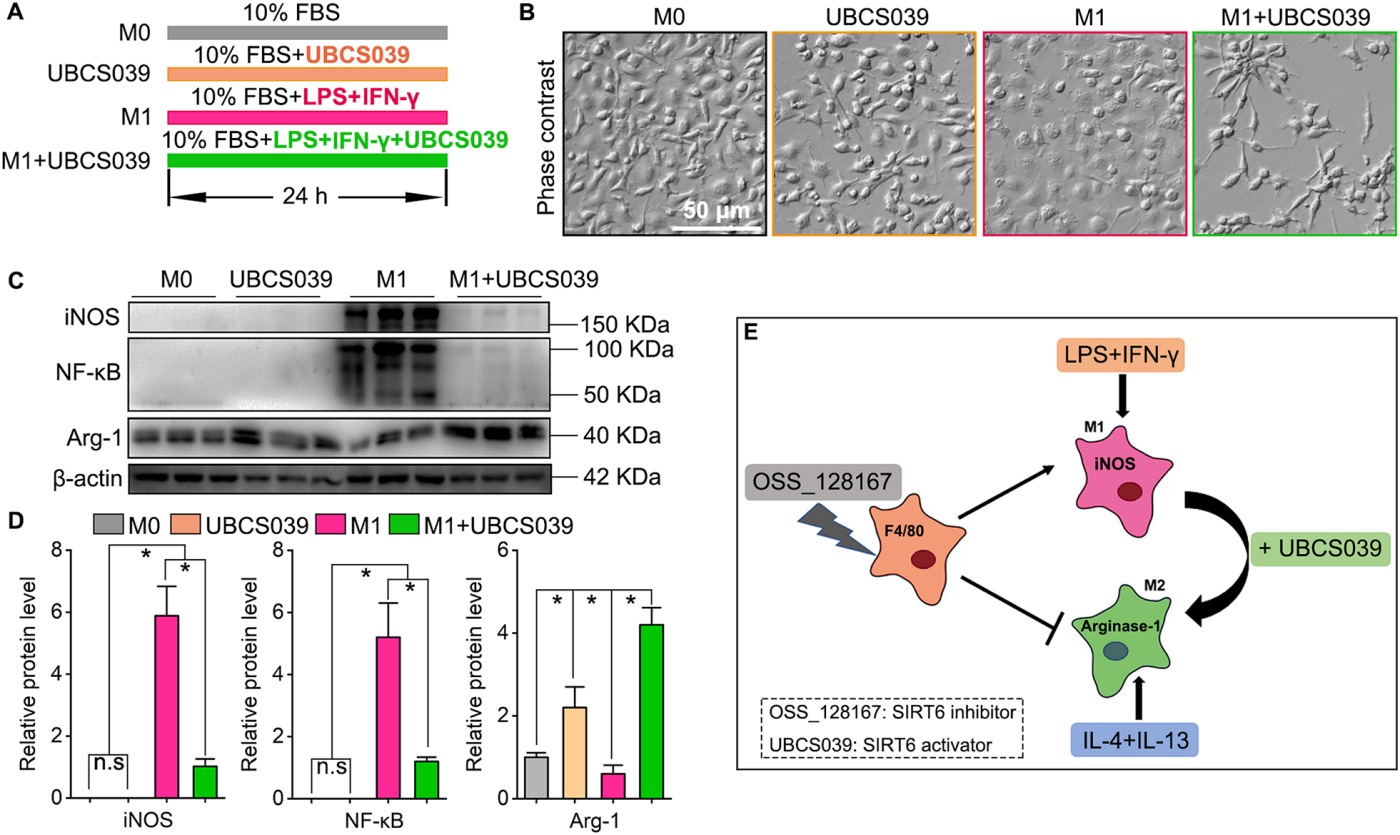
SIRT6 activation shifts the macrophages from M1 to M2 phenotype. **A** The cultured macrophages were stimulated with 150 μM UBCS039, or LPS and IFN-γ, and UBCS039 was added into culture medium in presence of LPS and IFN-γ for 24 h. **B** Cellular morphology of macrophages after stimulation. Scale bar: 50 µm. **C** Western blots of iNOS, NF-κB and Arg-1. **D** Quantification of iNOS, NF-κB and Arg-1 protein levels in macrophages. The protein levels were normalized with β-actin. *P  < 0.05. **E** Schematic diagram of how SIRT6 regulates M1/M2 polarization in macrophages

Next question is whether SIRT6 activation act on M1/M2 phenotype switch in macrophages. We added a selective SIRT6 activator UBCS039 into culture medium in presence of LPS and IFN-γ stimulation (Fig. 10A). From cellular morphology, we found that UBCS039 treatment can induce macrophages polarizing into M2 phenotype, and UBCS039 shift the macrophages from M1 to M2 phenotype forcefully (Fig. 10B). Western blots showed that UBCS039 markedly decreased iNOS and NF-κB expression but increased Arg-1 expression in macrophages (Fig. 10C, D). In summary, SIRT6 inhibition promotes M1 polarization but suppresses M2 polarization in macrophages, and SIRT6 activation shifts the macrophages from M1 to M2 phenotype (Fig. 10E).

## Discussion

SIRT6, a member of the sirtuin family of NAD^+^-dependent deacetylases, has been shown to produce beneficial effects in brain injury and spinal cord injury [21, 22]. However, the role of SIRT6 in macrophages involved peripheral nerve injury is still unclear. The new findings of the current study were as follows: (1) SIRT6 expression is significantly up-regulated in the injured nerve and its expression is accompanied with macrophages infiltration and macrophages phagocytosis, (2) SIRT6 silencing by shRNA lentivirus injection in sciatic nerve delays nerve regeneration and functional recovery, (3) and this adverse effect is probably due to the SIRT6 inhibition-mediated repress in macrophages migration, phagocytosis and M2-polarization of macrophages, but simultaneously increases M1-polarization of macrophages via activating TNF-α and NF-κB expression, (4) SIRT6 activation shifts M1 to M2 phenotype in macrophages. These findings highlight SIRT6, as a beneficial protein in ameliorating peripheral nerve injury, ensure migration, phagocytosis and M2-polarization of macrophages.

In mammalian and rodent, SIRT6 has been shown beneficial effects against various degenerative or inflammatory diseases. Recent studies illustrated that SIRT6 suppresses inflammatory responses in collagen-induced arthritis [23], and SIRT6 could inhibit TNF-α-induced inflammation in vascular adventitial fibroblasts [18]. Wu et al. reported SIRT6 overexpression significantly suppresses NF-κB activation and attenuates inflammatory responses in osteoarthritis [24]. Therefore, we speculate that SIRT6 might execute an essential role in peripheral nerve recovery. In the present study, we showed that SIRT6 expression is significantly up-regulated with macrophage infiltration in the injured sciatic nerve, and high expression level of SIRT6 was found during macrophage phagocytosis in vitro. Moreover, SIRT6 inhibition impairs phagocytosis capacity in macrophage. This findings reveal that SIRT6 participates in peripheral nerve repair partially via regulating macrophage phagocytosis, an important biological process during the early stage of nerve recovery [25]. Previous studies showed that SIRT6 execute opposite biological functions in the migration of diverse cell types. For instance, Chen et al. reported that SIRT6 inhibits migration of glioma cells via down-regulating Notch-3 expression [26], but it has reported that SIRT6 inhibition impairs migration of dendritic cell [27]. These studies suggested a complicated role of SIRT6 in cell migration. The present study showed SIRT6 inhibition significantly impairs migration of macrophages. Migration is a key cellular event of macrophages in the injured peripheral nerve, decreased number of macrophages will dramatically impair the recovery of peripheral nerve injury [1]. Therefore, decreased migration and phagocytosis of macrophages caused by SIRT6 inhibition may contribute to a repressive myelin debris removal and result in a disadvantageous microenvironment of regeneration. Furthermore, the delayed nerve regeneration and worse nerve conduction capacity suggest the potential role of SIRT6 on macrophages polarization, another essential biological process after nerve injury.

Upon peripheral nerve injury, a large number of macrophages are accumulated at the injury sites, where they not only contribute to axonal and myelin debris removal, but also are educated by the local microenvironment and polarized to an anti-inflammatory M2 phenotype, thus contributing to axonal regeneration [28]. We identified SIRT6 expression is decreased in M1 macrophages but increased in M2 macrophages, and SIRT6 inhibition promotes M1 macrophages polarization but reduces M2 macrophages polarization via activating NF-κB and TNF-α expression. The transcription factor NF-κB plays a key role in injury response by regulating genes involved in inflammation, and it has been reported that NF-κB pathway activation leading to an increase in the synthesis of pro-inflammatory cytokines TNF-α under high a level of reactive oxygen species [29]. Prior evidence suggested that SIRT6 prevents overactivation of the NF-κB pathway, not only by deacetylating H3K9 at the promoter of NF-κB target genes, but also by activating the NF-κB repressor, IκBα [30]. In addition, Thandavarayan et al. observed that SIRT6 knockdown impair diabetic wound closure with concomitantly increased levels of oxidative stress, inflammatory cytokines and NF-κB activation [31]. These findings explain that SIRT6 inhibition-induced inflammation response and increased M1 polarization in the present study. Besides, we found SIRT6 silencing up-regulate NF-κB and TNF-α expression and subsequently promote macrophages M1 polarization. Kawahara et al. reported that SIRT6 can inhibit NF-κB expression by deacetylating histone H3 lysine 9 to facilitate NF-κB destabilization [32]. And NF-κB is a well-known mediator of TNF-α, activation of NF-κB induce TNF-α expression and production [33]. Sufficient evidences point to a phenotypic switch from an M1 to an M2 macrophage is extremely important in the process of nerve injury repair. Huang et al. reported that the inhibition of M1 macrophage activation and the promotion of M2 macrophage transformation facilitate peripheral nerve regeneration [34]. In the present study, we found that SIRT6 activation inhibit NF-κB and TNF-α expression but increase Arg-1 expression, and shifts M1 to M2 phenotype in macrophages, our results suggest a beneficial role of SIRT6 on M2 polarization of macrophages and may encourage peripheral nerve recovery. Thus, in macrophages, SIRT6 promotes macrophages polarized into M2 type and mediated peripheral nerve regeneration. Our recent study has demonstrated SIRT6 acts as a negative regulator for Schwann cells dedifferentiation via c-Jun pathway during Wallerian degeneration in the injured nerve [35]. Which indicates that the SIRT6 inhibition in Schwann cells may also play roles in nerve regeneration. Therefore, we will use SIRT6 conditional knockout mice in Schwann cells or macrophages in the future to reveal the roles of SIRT6 in peripheral nerve regeneration and underlying mechanisms.

In summary, our data revealed that an indispensable role of SIRT6 in peripheral nerve repair via regulating macrophage functions including phagocytosis, migration and polarization. Although further molecular mechanism need to be explored, our study provided a promising target, SIRT6, to regulate the functions of macrophages and ensure the peripheral nerve repair.

## Materials and methods

### Experimental animals and surgery

Specific pathogen–free C57BL/6 mice (aged 8 weeks) weighing 20–22 g was provided by the Animal Center of Southern Medical University, China [License No. SCXK (Yue) 2016-0041]. The mice were housed in an animal room maintained at 21 ± 2 °C and 55% relative humidity with a 12-h light/12-hour dark cycle and given free access to water and food. Surgery of sciatic nerve transection was established as described in the previous paper [36]. Briefly, mice were anesthetized by intraperitoneal injection of 180 mg/kg tribromoethanol (Sigma-Aldrich, St. Louis, MO, USA). Then, the bilateral sciatic nerves were bluntly exposed, and transected by eye scissors in the site of 0.5 cm distal far from the sciatic notch, then closed the incision. Mice in the intact group were sham-operated (only undergoing nerve exposure). The sciatic nerves were collected for Western blotting analysis by decapitation, and immunofluorescence staining by transcardially perfused with 4% PFA, respectively. For crush injury of the sciatic nerve, the bilateral sciatic nerves were bluntly exposed, and a crush injury 0.5 cm distal to the sciatic notch was performed by clamping the nerve with a smooth, straight hemostat (tip width 2 mm) for 2 min [37].

### Electrophysiological examination

To assess nerve conduction of the injured nerve, the mice were anesthetized with 180 mg/kg tribromoethanol post 3 weeks after surgery, and electrophysiological examination was performed as previously reported [14, 37]. Briefly, a pair of needle electrodes were inserted to stimulate the sciatic nerve 3 mm proximal to the crush site, and a pair of recording electrodes were inserted subcutaneously into the middle of the intrinsic foot muscle. After a single stimulation with a strength of 10 mA and a duration of 0.1 ms, the amplitude and latency of the compound muscle action potential were recorded with a set of electrophysiological recorders (frequency of 20 Hz and pulse width of 0.1 ms) (Axon Digidata 1550 Digitizer, Molecular Devices, Sunnyvale, CA, USA). Immediately after the electrophysiological testing, the mice were transcardially perfused with 4% PFA, and the gastrocnemius muscles and sciatic nerves were collected for histomorphometry examination, Western blotting or immunofluorescence.

### Histomorphometry of the gastrocnemius muscle

To assess the level of myoatrophy of the target muscle of the injured nerve, the wet weight of the bilateral gastrocnemius muscles was recorded at 3 weeks after surgery. Then the mid-belly of the muscles was trimmed for routine paraffin embedding, transversal sectioning, and hematoxylin–eosin (H&E) staining to visualize the myofibers. Six non-overlapping images of every eighth section from each animal were captured, and the average myofiber area was quantified using Image J software (Media Cybernetics, USA).

### Culture of RAW264.7 and bone marrow-derived macrophages (BMDM)

The mouse monocyte-macrophage line RAW264.7 was purchased from the American Type Culture Collection (ATCC, USA) and maintained in Dulbecco’s modified Eagle medium (DMEM)/F12 (Cat#11330057, Corning, USA) supplemented with 10% fetal bovine serum (FBS, Cat#F8318, Sigma, USA), 100 mg/ml streptomycin, 100 U/ml penicillin (Cat#15140122, Gibco, USA) and incubated at 37 ℃ and an atmosphere of 5% CO_2_. Bone marrow-derived macrophages (BMDM) were isolated and cultured as previously described [38]. Briefly, bone marrow cells were flushed out with DMEM/F12 from the tibia and femur of mice (8 W). Then the cells were cultured for 7 days in DMEM/F12 containing 10% FBS and 10 ng/ml macrophage colony stimulating factor (MCSF, Cat#416-ML-010, R&D systems, USA) at 37 °C and under 5% CO_2_. The BMDM was identified by immunofluorescence for macrophage marker F4/80 staining before further experiments.

### Treatment of SIRT6 inhibitor and calcein-AM/propidium iodide (PI) staining

To investigate the role of SIRT6 in migration, phagocytosis and polarization of macrophages, the SIRT6 selective inhibitor OSS_128167 (Cat#HY-107454, MedChemExpress, USA) was used to suppress the activity of SIRT6 [39]. And to assess the potential cytotoxicity of OSS_128167, Calcein-AM/ Propidium Iodide (PI) staining assay was performed by using a Live/Dead assay kit (Cat#BB-4126, BestBio, Shanghai, China). Briefly, BMDM were seeded at 2 × 10^4^ per well in 24-well plates and incubated overnight to allow cell adherence. Then treated with 200 μM of SIRT6 inhibitor OSS_128167 for 24 h based on the previous studies [39, 40], and the BMDM treated with 0.1‰ DMSO was assigned as the Vehicle group. The cells were washed once with sterile PBS and incubated in 0.4 μM fluorescent green Calcein-AM for 30 min at 37 ℃ to check live cells and 2 μM fluorescent red PI for 5 min at 37 ℃ for dead cells.

### Lentivirus-mediated shRNA transfection

ShRNA lentivirus were constructed by Genechem Corporation (Shanghai, China). A 58-nucleotide sequence, named as shSIRT6: 5′-CCGGGCATGTTTCGTATAAGTCCAACTCGAGTTGGACTTATACGAAACATGCTTTTTG-3′, corresponding to the targeted SIRT6 mRNA was selected and constructed. 5′-TTCTCCGAACGTGTCACGT-3′ was used as the controlled sequence and named as shControl. Lentiviruses were produced using 293 T cells, and the viral titers of shControl and shSIRT6 reached 4 × 10^8^ and 5 × 10^8^ TU/ml, respectively. For lentivirus transfection in vivo, the lentivirus-vector was microinjected into the sciatic nerve as previously described [41, 42]. Mice were deeply anesthetized and the sciatic nerves were exposed and crushed, immediately 2.5 μl of shControl or 2 μl of shSIRT6 was injected inside the bilateral crushed nerve using a glass capillary with 80 mm diameter tip under a dissection microscope (Cat#GD200, ZOUSUN Optical Instrument, Shanghai, China). Fast green (Cat#F7252, Sigma, USA) was added to the viral vector solutions at a final concentration of 0.2% to visualize the spread of the solution during injection. The wound was sutured and disinfected. 3 weeks after the injection, the sciatic nerves of three animals per group were harvested for analysis. For lentivirus transfection in vitro, RAW264.7 or BMDM were incubated with DMEM/F12 containing 10% FBS at a multiplicity of infection (MOI) of 10 for 24 h. After discarding the lentiviruses, the cells were cultured for 48 h further before assessments. In order to identify the effect of RNA interference, the SIRT6 expression in the transfected nerves or cells was detected by Western blotting.

### In vitro migration assay

The migration of BMDM was assessed using 6.5-mm Transwell chambers with 8-μm pores (Cat#3422, Corning Costar, USA). After equilibrating the membrane with 600 μl of DMEM/F12 containing 10% FBS in the lower chamber and 100 μl of DMEM/F12 containing 1% FBS in the upper chamber overnight. The cells from each group were seeded at 1 × 10^5^/ml and allowed to migrate at 37 °C in 5% CO_2_ for 20 h and then fixed by 4% PFA for 30 min. After careful removal of cells on the upper surface with a cotton swab, the cells adhering to the lower-bottom surface of each membrane were stained with Crystal Violet (Cat#0121, Beyotime Biotechnology, Wuhan, China). All assessments were repeated three times by using three chambers for each test. Then, images were captured under an inverted microscope (Leica, Germany) and the number of migrated cells was quantified in five 200 μm × 200 µm fields. Cell migration was also assessed by a two-dimensional scratch assay. Briefly, BMDM (2 × 10^6^/ml) were grown in a 6-well plate and treated with 200 μM of SIRT6 inhibitor OSS_128167 or 0.1‰ DMSO for Vehicle group. After 24 h, the cells in the center of the well were scratched with a 200-μl sterile pipette tip to create a cell-free area. The scratched area was photographed using an inverted microscopy (Leica, Germany) soon after scratching for 4 h and 8 h later. The scratch area was measured using Image J software (Media Cybernetics, USA), and the relative healing area was calculated based on the previous study [43].

### Phagocytosis assay

Myelin debris was produced as previously reported [44]. Briefly, the brain and spinal cord were obtained from adult mice after euthanasia, and then shattered into tiny particles by sonication. The debris was rinsed with double distilled water for three times by centrifugation for 15 min at 4 ℃ at 13,000×*g*. Finally, myelin debris was re-suspended with Hank’s balanced salt solution (HBSS, Cat#C14175500BT, Gibco, USA) at a concentration of 100 mg/ml and stored at − 80 ℃ until use. For detecting the phagocytosis capacity of macrophages after inhibiting SIRT6, BMDM were seeded in 24-well plates at a density of 1 × 10^5^ cells/well and treated with OSS_128167 or transfected with shRNA lentivirus, then 0.1 mg/ml fluorescent lumispheres (1 µm diameter, Cat#7-3-0100, BaseLine Chromtech, China) or 1 mg/ml myelin debris were added into the culture for 4 h, respectively. After being rinsed three times with HBSS to remove the attached lumispheres or myelin debris from the cell surface, the cells were fixed with 4% PFA and stained with F4/80 (1:400, Cat#ab6640, Abcam, USA) to identify the outline of macrophages. To observe the ingestion of myelin debris, F4/80 was co-stained with Oil red O (ORO) that marked as lipid droplets [37]. Briefly, the 0.3% ORO staining solution was prepared by mixing ORO (powders dissolved in 60% isopropanol) with deionized water (ratio  = 3:2). BMDM were rinsed in 0.01 M phosphate buffered saline (PBS) and 60% isopropanol, then incubated in the ORO solution for 40 min at room temperature and rinsed in 60% isopropanol and 0.01 M PBS and mounted.

### Polarization of RAW264.7 and BMDM

M1-type macrophages were induced by treatment with LPS (100 ng/ml) and IFN-γ (20 ng/ml) in the cell medium of DMEMF/12 containing 10% FBS for 24 h. M2-type macrophages were induced by treatment with IL-4 (20 ng/ml) and IL-13 (20 ng/ml) in the cell medium of DMEMF/12 containing 10% FBS for 24 h, as previously reported [45]. To study the effect of SIRT6 inhibition in macrophages polarization, 200 μM OSS_128167 was added into M1-stimulated medium or M2-stimulated medium. To investigate whether SIRT6 activation affects M1/M2 phenotype shift in macrophages, 150 μM SIRT6 activator UBCS039 (Cat#HY-115453, MedChemExpress, USA) was added into M1-stimulated medium for 24 h.

## RNA extraction and quantitative real-time PCR (qPCR)

Total RNA was isolated using Trizol (Invitrogen) method according to the manufacturer’s protocol. And the concentration of the RNA was determined by Nano-Drop system (Thermo, USA). Then cDNA was generated from 2 μg of total RNA by ReverTra Ace qPCR RT Kit (Cat#FSQ-101, Toyobo, Japan), and the transcripts were amplified and detected by quantitative real-time PCR in a Bio-Rad CFX machine (Bio-Rad, USA) using SYBR green (Cat#FP205, TIANGEN, Beijing, China). *GAPDH* was used as the internal reference. Primers were used as follows: *SIRT6* forward: 5′- TGACACCACCTTCGAGAATGCT-3′, *SIRT6* reverse: 5′-AGACAAATCGCTCCACCAAC-3′; *iNOS* forward: ATTCACAGCTCATCCGGTACG, *iNOS* reverse: GGATCTTGACCATCAGCTTGC; *Arg-1* forward: CTCCAAGCCAAAGTCCTTAGAG, *Arg-1* reverse: AGGAGCTGTCATTAGGGACATC; *GAPDH* forward: 5′-AGTGCCAGCCTCGTCCCGTAGACAA-3′, *GAPDH* reverse: 5′-CAGGCGCCCAATACGGCCAAAT-3′. The ratio of the relative expression for each gene to *GAPDH* was calculated by using the 2^−ΔΔCT^ formula.

### Immunofluorescence staining

The transected sciatic nerves were collected at 3 and 5 days after surgery, and fixed in 4% PFA for 24 h and dehydrated in 30% sucrose overnight. Then they were embedded in optimal cutting temperature (OCT; Sakura Finetek, Torrance CA, USA) for cryosectioning. The 10 µm—thick cryosection slices or cells were permeabilized with 0.5% Triton X-100 (Sigma) for 1 h, then blocked with 5% fish gelatin (Sigma) containing 0.3% Triton X-100 at room temperature for 1 h, followed by incubation with the primary antibodies overnight, then incubated with Alexa-conjugated (568 or 488) secondary antibodies for 2 h at room temperature. The samples were then incubated with 4′,6-diamidino-2-phenylindole (DAPI; 1:2000; Sigma, USA) for 5 min to counterstain the cell nuclei. The primary antibodies used for immunofluorescence were as follows: rat anti-F4/80 (1:400, Cat#ab6640, Abcam, USA), rabbit anti-SIRT6 (1:50, Cat#12486S, Cell Signaling Technology, USA), rabbit anti-iNOS (1:400, Cat#ab178945, Abcam, USA), rabbit anti-CD163 (1:200, Cat#ab182422, Abcam, USA). For quantification analysis, five pictures from random field were taken and the number of cells labeled positive for the different markers was determined in reference to the total number of cells (DAPI staining) in a 400 × 400 μm area. For detection of morphological changes, cells were plated, grown on coverslips and imaged under a Leica inverted phase-contrast microscope (Leica, Germany).

### Western blotting

Protein extracts from tissues or cells were lysed in RIPA lysis buffer (containing 50 mM Tris (pH  = 7.4), 150 mM NaCl, 1% TritonX-100, 1% sodium deoxycholate, 0.1% SDS, sodium orthovanadate, sodiumfluoride, EDTA and eupeptin) (Cat#FD009, FUDE Biological Technology, Hangzhou, China). The lysates were centrifuged at 13,000×*g* for 30 min at 4 °C, and the supernatant was collected. The protein lysates were then separated on 10% dodecyl sulfate sodium salt-polyacrylamide gel electrophoresis gels and transferred to polyvinylidene fluoride membranes (Bio-Rad, Hercules, CA, USA). After blocking with 5% skim milk (Cat#D8340, Solarbio Life Sciences, Beijing, China) in Tris-buffered saline (TBS, 25 mM Tris–HCl pH 7.5, 140 mM NaCl) containing 0.5% Tween-20 (TBST) for 2 h at room temperature, the blots were incubated overnight at 4 °C with the following primary antibodies dissolving in QuickBlock™ Primary Antibody Dilution Buffer (Cat#P0256, Beyotime Biotechnology, Shanghai, China): rabbit anti-SIRT6 (1:1000, Cat#ab62739, Abcam, USA), rat anti-F4/80 (1:500, Cat#ab6640, Abcam, USA), mouse anti-MAG (1:1000; Cat#ab89780, Abcam, USA), mouse anti-MBP (1:500, Cat#SMI-99, BioLegend, California, USA), mouse anti-β-actin (1:2000, Cat#ab008, Multi Sciences, Hangzhou, China), rabbit anti-myelin protein zero (MPZ, 1:1000, Cat#ABN363, Sigma-Aldrich, USA), mouse anti-c-Jun (1:500; Cat#610326, BD Biosciences, USA), rabbit anti-iNOS (1:1000, Cat#ab178945, Abcam, USA), rabbit anti-TNF-α (1:1000, Cat#ab205587, Abcam, USA), rabbit anti-CD163 (1:1000, Cat#ab182422, Abcam, USA), and rabbit anti-Arginase-1 (Arg-1, 1:500, Cat#93668S, Cell Signaling Technology, USA). After three 30 min washes with TBST, the blots were incubated with horseradish peroxidase (HRP)-conjugated anti-rabbit secondary antibody (1:2000, Cat#65-6120, Molecular Probes, USA), anti-mouse secondary antibody (1:2000, Cat#SA5-10317, Invitrogen, USA) or anti-rat secondary antibody (1:1000, Cat# SA5-10278, Invitrogen, USA) for 2 h at room temperature, and were visualized using enhanced chemiluminescence (Cat#SQ101, EpiZyme, Shanghai, China). Finally, the density was calculated using Image J software (Media Cybernetics, USA). The relative protein expression levels were normalized to β-actin.

### Statistical analysis

All data are presented as mean  ±  standard error of the mean (SEM). All statistical analyses were performed with Origin 8.0 software (OriginLab, Northampton, Massachusetts, USA) using one-way analysis of variance followed by Bonferroni’s multiple test. The two-tailed Student’s *t *test for normally distributed data with comparable variances was used to highlight statistical differences between the two groups. A *P* value of  < 0.05 was considered statistically significant.

## Data Availability

The datasets used and/or analysed during the current study are available from the corresponding author on reasonable request.

## Abbreviations

SIRT6: Silent information regulator 6
BMDM: Bone marrow-derived macrophages
Arg-1: Arginase-1
